# Supplementary material for: Pharmacokinetics of metamizole (dipyrone) as an add-on in calves undergoing umbilical surgery
Source: PLoS One. 2022 Mar 15;17(3):e0265305. doi: 10.1371/journal.pone.0265305 (PMC8923478; doi:10.1371/journal.pone.0265305)
Supplement: Attachment — Submitted filename: Response to Reviewers.docx [file pone.0265305.s001.docx]

Journal Requirements:

When submitting your revision, we need you to address these additional requirements.

1. Please ensure that your manuscript meets PLOS ONE's style requirements, including those for file naming. The PLOS ONE style templates can be found at

https://journals.plos.org/plosone/s/file?id=wjVg/PLOSOne_formatting_sample_main_body.pdf and

https://journals.plos.org/plosone/s/file?id=ba62/PLOSOne_formatting_sample_title_authors_affiliations.pdf

2. In your Methods section, please provide additional details regarding the animals used in your study and ensure you have described the source. For more information regarding PLOS' policy on materials sharing and reporting, see https://journals.plos.org/plosone/s/materials-and-software-sharing#loc-sharing-materials.

For your better understanding we would like to give you the following information:

The experimental animals were selected after a detailed initial veterinary examination. Inclusion criteria were a simple umbilical hernia worthy of surgery with otherwise clinically unremarkable findings and an undisturbed general condition of the animal. They also had to be at least 4 weeks old and no more than 3 months old. Exclusion criteria were reduced general condition, additional suffering from a disease such as diarrhoea or pneumonia, and receipt of analgesic or antiphlogistic therapy within the last 48 hours before the start of the trial. No consideration was given to race or sex. The criterion for termination during the experiment was a massive deterioration in the general condition of the animals (according to score sheet of the authority approval) or a disturbance in the timing of the experiment.

All calves came from agricultural livestock farms in Bavaria (Federal State in Southern Germany). The animals were purchased by the clinic at calf markets in the surrounding area (e.g. Miesbach).

To provide more detailed information in the manuscript we expanded the part „Animals“ in the M+M section as follows:

 Eight Simmental calves (one female, seven males) were purchased by the clinic from a local livestock market (Zuchtverband Miesbach, Germany) for this study. At the day of purchase a short clinical examination by a veterinarian showed no further health disorders apart from uncomplicated umbilical hernia. The average age of the animals was 47.6 ± 10.4 days, and the average body mass was 90 ± 10.8 kg. In the Clinic for Ruminants of the Ludwig-Maximilians University of Munich (Germany), the animals were housed in individual pens (igloos) with hearing, olfactory and visual contact with conspecifics at all times. The experimental animals were strictly isolated from the regular clinic patients by spatial and hygienic measures. The calves were fed whole milk three times a day and had free access to water, hay, a total mixed ration, and mineral licks. The calves stayed at the clinic at least 6 days before surgery. The inclusion criteria were based on clinically unremarkable findings by a veterinarian and an undisturbed general condition of the animal incl. feed intake, and blood analysis (CBC without a differential) one day before surgery. The exclusion criteria were any signs of disease other than an uncomplicated hernia. No complications (fever, peritonitis, surgical incisional infection) occurred during the study, and the animals were sold afterwards to a local cattle dealer. This was possible as only substances, which are listed as allowed substance for food producing animals in the European Commission Regulation (EU) No 37/2010, were applied to the calves. Therefore, slaughtering was still possible after awaiting at least the mandatory waiting period for consumable tissue, that is listed in the European Commission Regulation above for each allowed substance.

3. In your Methods section, please state the volume of the blood samples collected for use in your study.

The sentence was expanded as follows:

Before each blood sampling, the IV catheter was flushed with a 0.9% sodium chloride solution and 2 mL of blood was withdrawn and discarded. Subsequently, 2 mL of EDTA blood were taken and serum was isolated by centrifugation of the blood samples (10 min, 3363 x g) and stored at -70 °C until further analysis.

4. We noted you reported at line 105 "the animals were sold afterwards to a local cattle dealer". As we understand that the animals were sold for food purposes at the end of the study, please confirm whether this was approved by the relevant national and/or local Food Safety Administration.

 Yes, this is legal in Germany /within the European Union when only substances, which are listed as allowed substance for food producing animals in the European Commission Regulation (EU) No 37/2010, were applied to the calves. Therefore, slaughtering was still possible after awaiting at least the mandatory waiting period for consumable tissue, that is listed in the European Commission Regulation above for each allowed substance:

- Metamizole: due to Table 1 of the Commission Regulation (EU) No 37/2010, the current Maximum Residual Limit (MRL) for metamizole in food producing cattle is 100 µg/kg each in fat, liver, muscle and kidneys, respectively, and 50 µg/kg in milk. The latency in cattle before slaughtering is 12 days for consumable tissue (IV) and 4 days for the dispensing of milk (IV), respectively.
- Xylazine: due to Table 1 of the Commission Regulation (EU) No 37/2010, no MRL in food producing animals is required. The latency in cattle before slaughtering is 3 days for consumable tissue and 3 days for the dispensing of milk, respectively.
- Ketamine: due to Table 1 of the Commission Regulation (EU) No 37/2010, no MRL in food producing animals is required. The latency in cattle before slaughtering is 3 days for consumable tissue.
- Meloxicam: due to Table 1 of the Commission Regulation (EU) No 37/2010, the current Maximum Residual Limit (MRL) in food producing cattle is 20 µg/kg in muscle, 65 µg/kg each in liver and kidneys, respectively, and 15 µg/kg in milk. The latency in cattle before slaughtering is 15 days for consumable tissue (IV) and 5 days for the dispensing of milk (IV), respectively.
- Isoflurane: due to Table 1 of the Commission Regulation (EU) No 37/2010, no MRL is required. The latency before slaughtering in horses and swine is 2 days for consumable tissue. Cattle are not explicitly authorised, but can be reclassified in accordance with the reclassification cascade in the Medicines Act for food-producing animals*.

(* In Germany, a therapeutic emergency is regulated by Section 56a (2) of the German Medicines Act (AMG). Here, medicines may be used in animals even if they do not have a marketing authorization for the respective animal species or indication, provided that there is no risk to human or animal health. For food-producing animals, it is also mandatory that the active substance is listed in Table 1 of the Commission Regulation (EU) No 37/2010 or is one of the so-called "out of scope" substances. For reclassification, i.e., the use of a drug for another species or indication, veterinary law prescribes a "reclassification cascade": First, another drug must be used that is approved for the particular species but not for the indication, if that drug also promises a therapeutic effect. For food-producing animals, the appropriate waiting period must be observed. If such a drug is not available, one approved for another species may be used. The latency is set at a minimum of 28 days for consumable tissues, 10 days for eggs, and 7 days for milk.)

From this follows: As isoflurane was reclassified from other food-producing species, calves could earliest have been sold for slaughtering 28 days after anesthesia.

5.Thank you for stating the following in the **Financial Disclosure** section:

"Magdalena Behrendt-Wippermann got a grant  from the H. Wilhelm Schaumann Stiftung Germany (https://www.schaumann-stiftung.de/de/forderung-1764.htm).  The study was additionally supported by Richter Pharma AG, Austria (https://www.richter-pharma.at/).
The funders had no role in study design, data collection and analysis, decision to publish, or preparation of the manuscript."

 We note that you received funding from a commercial source: Richter Pharma AG

Please provide an amended Competing Interests Statement that explicitly states this commercial funder, along with any other [relevant declarations](about:blank) relating to employment, consultancy, patents, products in development, marketed products, etc.

Within this Competing Interests Statement, please confirm that this does not alter your adherence to all PLOS ONE policies on sharing data and materials by including the following statement: "This does not alter our adherence to PLOS ONE policies on sharing data and materials.” (as detailed online in our guide for authors [http://journals.plos.org/plosone/s/competing-interests](about:blank)).  If there are restrictions on sharing of data and/or materials, please state these. Please note that we cannot proceed with consideration of your article until this information has been declared.

 Please include your amended Competing Interests Statement within your cover letter. We will change the online submission form on your behalf.

Please know it is PLOS ONE policy for corresponding authors to declare, on behalf of all authors, all potential competing interests for the purposes of transparency. PLOS defines a competing interest as anything that interferes with, or could reasonably be perceived as interfering with, the full and objective presentation, peer review, editorial decision-making, or publication of research or non-research articles submitted to one of the journals. Competing interests can be financial or non-financial, professional, or personal. Competing interests can arise in relationship to an organization or another person. Please follow this link to our website for more details on competing interests: [http://journals.plos.org/plosone/s/competing-interests](about:blank)

This is our amended Competing Interests Statement including explanations:

Magdalena Behrendt-Wippermann got a grant from the H. Wilhelm Schaumann Stiftung Germany (<https://www.schaumann-stiftung.de/de/forderung-1764.htm>), and the study was additionally supported by Richter Pharma AG, Austria (<https://www.richter-pharma.at/>). This company generally produces a metamizole injection solution for animals (Novasul^®^ 500 mg/ml for horses, cattle, swine and dogs). When we looked for financial support for the study, Richter Pharma AG sponsored us with a one-off payment without any terms. They did not even demand that we had to use their Austrian product in the study, we used a German one. Therefore, the funding sources had no involvement in the study design, collection, analysis and interpretation of data, the writing of the report and in the decision to submit the article for publication. None of the authors of this paper have any financial or personal relationships with other people or organisations that could inappropriately influence or bias the content of the paper. There were no conflicts of interest due to employment, consultancy, patents, products in development, marketed products etc., and there are no restrictions on sharing of data and/or materials. This does not alter our adherence to PLOS ONE policies on sharing data and materials.

[Note: HTML markup is below. Please do not edit.]

Reviewers' comments:

Reviewer's Responses to Questions

**Comments to the Author**

1. Is the manuscript technically sound, and do the data support the conclusions?

The manuscript must describe a technically sound piece of scientific research with data that supports the conclusions. Experiments must have been conducted rigorously, with appropriate controls, replication, and sample sizes. The conclusions must be drawn appropriately based on the data presented.

Reviewer #1: Partly

Reviewer #2: Partly

2. Has the statistical analysis been performed appropriately and rigorously?

Reviewer #1: No

Reviewer #2: I Don't Know

3. Have the authors made all data underlying the findings in their manuscript fully available?

The [PLOS Data policy](http://www.plosone.org/static/policies.action#sharing) requires authors to make all data underlying the findings described in their manuscript fully available without restriction, with rare exception (please refer to the Data Availability Statement in the manuscript PDF file). The data should be provided as part of the manuscript or its supporting information, or deposited to a public repository. For example, in addition to summary statistics, the data points behind means, medians and variance measures should be available. If there are restrictions on publicly sharing data—e.g. participant privacy or use of data from a third party—those must be specified.

Reviewer #1: No

Reviewer #2: Yes

4. Is the manuscript presented in an intelligible fashion and written in standard English?

PLOS ONE does not copyedit accepted manuscripts, so the language in submitted articles must be clear, correct, and unambiguous. Any typographical or grammatical errors should be corrected at revision, so please note any specific errors here.

Reviewer #1: No

Reviewer #2: Yes

5. Review Comments to the Author

Please use the space provided to explain your answers to the questions above. You may also include additional comments for the author, including concerns about dual publication, research ethics, or publication ethics. (Please upload your review as an attachment if it exceeds 20,000 characters)

**Reviewer #1:** Pharmacokinetics of metamizole (dipyrone) used as an add on analgesic in calves undergoing umbilical surgery. Fux D, et al., 2020

Interesting study with a combination of analgesic drugs, other drugs. There is a disconnect between the actual study and the description of similarities and differences between models. Much to consider. My suggestion is to revise (shorten) to make flow more appropriate. If you meant to conduct PK-PD study, I think it missed the mark. If you want to conduct PK study in calves, describe this study. Some attention to possible implications may be helpful, but limit to ruminant or monogastric etc.
Thank you very much for your assessment. We have thoroughly revised the manuscript again and have tried very hard to take up these suggestions.

Abstract –
The first line of the abstract states: The pharmacokinetic behaviour of metamizole (dipyrone) and its metabolites 4-MAA, 4-AA was evaluated in this clinical, veterinary research study in calves…
While the statements below indicate only 4-MAA and 4-AA were followed (based upon rapid conversion of metamizole to metabolites). However, it does not appear plasma concentration of parent drug were followed. Please revise to make this correct.

Thank you, we revised the abstract completely:

This preliminary clinical investigation of the pharmacokinetic behavior of the main metamizole (dipyrone) metabolites 4-methylaminoantipyrine (4-MAA) and 4-aminoantipyrine (4-AA) in calves undergoing umbilical surgery was part of an already published main study. A single intravenous dose of metamizole was added to ketamine/xylazine/isoflurane anesthesia. (…)

Line 34. “time point 15 minutes after application.” If the drug was topically applied, application is an appropriate term. If given parenterally, administration/injection/IV catheter etc is likely more appropriate.

Thank you for the comment. We changed the term to „administration“.

Line 36-39. Intriguing finding of different disposition for 4-AA. Please expand upon this information?

Thank you, we revised the abstract completely:

(…) In contrast, 4-AA either showed a fast, high and biphasic behavior in five calves (C_max 15-30min_ 2.54-2.66 µg/mL and C_max 2-3.5h_ 2.10-2.14 µg/mL) with an elimination half-life of 8.87 hours, indicating a rapid distribution and subsequent redistribution from well-perfused organs, what effects a second increase of plasma concentration. Or, three calves exhibited a slower and lower monophasic increase (C_max 6.5h_ 1.65 µg/mL) with an elimination half-life of 6.23 hours, indicating a slow accumulation in the intravascular compartment. (…)

Line 42-48. You indicate that metamizole is converted to 4-MAA and 4-AA in anesthetized calves. You then state 4-MAA follows 2-compartment model with rapid distribution and slower elimination phases. They you state that kinetic data support the clinical analgesic effect of observed there. You do not provide any data to support the analgesic effects of metamizole? Please clarify how Tmax, Cmax, for metabolites demonstrates analgesic effects?

Thank you, we revised the abstract completely:

(…) That metabolic behavior support our already published data on clinical monitoring and plasma cortisol levels (PCC). Compared to saline controls, lower PCC levels correspond to the distribution half-life of 4-MAA. Data of T_max_ and elimination half-life also match with those clinical observations. However, further studies are required to assess the exact analgesic mechanism and potency of the metamizole metabolites in calves.

Please let us more explain our conclusion for you:

The idea to investigate this study primarily was to improve the intraoperative analgesia for the calves by add-on metamizole to our standard anesthetic protocol in the Clinic for Ruminants of the Ludwig-Maximilians University of Munich (Germany). First of all, we looked at the clinical monitoring parameters intraoperatively, if changes could be detected with or without metamizole add-on. To better verify the unconscious nociceptive reaction to painful stimuli during surgery, we additionally measured plasma cortisol levels as an indicator of nociception. These data are already published [26]. Accordingly, our first publication included data from 26 animals plus the control animals without metamizole (saline group). From 8 animals of the overall study, we additionally had the metamizole metabolites 4-MAA and 4-AA determined as a guide. These animals are the subject of this manuscript. With this orientating number of animals, we additionally wanted to get a first overview of the metabolic behaviour of metamizole in calves (especially the maximum plasma concentration of its main metabolites and the elimination half-life) under the same clinical (field) conditions. With our “PK data” we cannot directly say that Tmax/Cmax demonstrates the analgesic effect of metamizole. However, to these 8 animals also plasma cortisol soldiers exist.

Due to the word limit of max. 300, we have not further listed these results in the abstract (as they are also already published). In the discussion, however, we mentioned some of those results more detailed:

(…) Briefly summarized, the already published [26] plasma cortisol concentrations (PCC) showed no significant difference in baseline values before surgery (p = 0.84). During surgery a general cortisol release could be observed in all animals, but compared to saline controls (CG), the increase of PCC was delayed and consistently lower in metamizole-treated calves (MG). Afterwards, PCC remained elevated in the CG, whereas at 150 minutes after skin incision a significant decrease was observed for MG (MG: 11.6 (8.4 - 16.5) versus CG: 39.1 (27.3 - 81.4) nmol/L; p = 0.0026). Overall, the mean PCC in MG was 10.9 nmol/L lower than that in CG (p = 0.01). After 8.5 hours, cortisol levels equaled again in both groups (CG: 19.71 ± 16.51 nmol/L; MG: 15.98 ± 7.88 nmol/L). These results fit with the observed preliminary metabolic data (elimination half-life and C_max_ of 4-MAA and 4-AA) of metamizole. The mentioned general cortisol release probably had non-pain related reasons as isoflurane, xylazine, and also the placement of calves in dorsal recumbency may elevate plasma cortisol level [2,30,31]. (…)

(…) Our already published clinical monitoring data [26] showed that mean arterial pressure (MAP) significantly increased during anesthesia, independent of metamizole administration. This may have resulted from a waning in the hypotensive action of xylazine [58]. Heart rate (HR) initially decreased in almost parallel courses with and without metamizole until the beginning of the ‘suturing of the peritoneum and fascia’ period (29 minutes after skin incision), probably related to the depressive casrdiovascular effect of ketamine-xylazine, before it remained relatively constant until end of surgical intervention with metamizole add-on. The CG in contrast showed an increase afterwards.

Line 62-63. Please clarify how one can tease the 4-MAA, 4-AA effects of metamizole from the systemic effects of meloxicam (co-administered to calves)? Did the meloxicam produce changes in disposition of 4-MAA, 4-AA or is there a pharmacogenetic component in these animals?

Thank you for your comment. Clarifying this aspect would be very speculative as we have not directly investigated any pharmacokinetic interferences between meloxicam and metamizole in our study, and we have no control group with pure metamizole without meloxicam.

Due to the word limit of max. 300, we have not further mentioned that speculative aspect in the abstract. In the discussion, however, we speculated about it and discussed it compared to literature references:

(…) In humans, both metamizole metabolites are known as direct and reversible COX inhibitors with an IC50 of 2.55 μM and 4.65 μM for COX1 and COX2 by 4-MAA, respectively, and 8.2- to 9-fold higher IC50 values for 4-AA [48]. In our calves, only plasma concentration of 4-MAA, but not 4-AA, was above that described COX-1/2-relevant IC50 values over a period of 24 hours.

But whether COX-inhibition by 4-MAA is the only analgesic mechanism of metamizole is unclear. Lower PCC levels of the 5 biphasic 4-AA metabolizers may also suggest analgesia by 4-AA in respective calves. Because 4-AA concentration is not sufficient for COX inhibition, an alternative mechanism must account for postoperative analgesia. After distribution into brain and spinal cord, 4-AA may be transformed into CNS-restricted metabolites, which are potent regulators of the endocannabinoid and vanilloid system [49,50]. As both systems modulate inflammatory nociception [51,52], CNS-distributed 4-AA might be relevant for enhanced suppression of perioperative pain in those animals.

While an analgesic effect of 4-MAA by inhibition of prostaglandin synthesis is likely in calves, an interference with pre-applied COX inhibitor meloxicam is negligible as meloxicam inhibits COX activity by competing with the arachidonic acid binding side, whereas 4-MAA by sequestering COX stimulating radicals [53]. As 4-MAA generation occured within 15 minutes after application, pre-applied meloxicam did not prevent 4-MAA synthesis in calves in principle. The non-steroidal anti-inflammatory drug (NSAID) meloxicam was primarily given to produce post-operative analgesia as post-operative pain results from prostaglandin-induced inflammatory processes in response to surgical trauma [54]. Although it was already applicated pre-emtively, a sufficient release of surgery-related inflammatory pain by NSAIDs is not clear [2]. (…)

(…) Compared to adult cattle, however, 4-AA synthesis was overall slower in calves, which may be caused by immature activity of the two enzymes of the cytochrome P450 monooxygenase superfamily CYP2B and CYP3A [38]~~[31]~~, or substrate competition, as ketamine and xylazine are also CYP3A substrates [39]~~[32]~~. Human data show that meloxicam, or rather its 5-hydroxylation metabolite, is predominantly catalyzed by CYP2C9, with only minor contribution of CYP3A4 [40]~~[33]~~. Attributable activity and concentration differences in these biotransformation enzymes in the liver, specifically CYP2C9, have been identified in sheep, cattle, and goats [41]~~[34]~~. Generally, CYP enzymes are species-specific. In cattle, tissue-specific mRNA expression of different cytochrome P450 (CYP) isoforms could be proved. The absolute quantification of liver mRNAs showed that CYP3A38 was the most abundantly expressed CYP3A isoform in bovine liver, followed by CYP3A48. Conversely, CYP3A28 (corresponding to the abundant human CYP3A4) was expressed at levels <1% in different cattle breeds. Likewise to humans, physiological factors such as age, gender and breed have been shown to affect bCYP3A expression and/or activity [42]. (…)

Line 70-71. Labelled for humans, cats, dogs, horses, cattle, swine. From the US FDA (https://www.fda.gov/animal-veterinary/product-safety-information/zimeta-dipyrone-injection-veterinarians), the following is stated:
Zimeta belongs to the pyrazolone class of non-steroidal anti-inflammatory (NSAID) drugs and is the first injectable dipyrone product to receive FDA approval for use in horses. Zimeta is for use in horses only. Zimeta has not been evaluated for use in horses intended for human consumption or food-producing animals, including lactating dairy animals.
Zimeta is not for use in humans. Direct contact with the skin should be avoided. Precautions should be employed by practitioners when handling and using loaded syringes to prevent accidental self-injection, as epidemiological studies have indicated that dipyrone can cause agranulocytosis in humans.
Perhaps this should be clarified (the FDA product safety information) is specific to the USA. They also use language such as: “Dipyrone is now prohibited for use in humans in several countries.” Similar language may clarify this difference in countries where the use is not controlled as much?

After Reviewer 2 also wanted us to mention that this drug has been associated with agranulocytosis in humans and has been banned for use in food animals at various times in recent years in some countries, we increased this part in the introduction as follows:

(…) In human medicine it was already launched in 1920 by the German Hoechst AG. But especially the rare risk of reversible but potentially fatal agranulocytosis led to the introduction of compulsory prescriptions for metamizole in Germany. Today, risk assessment and evaluation vary considerably from country to country. In many parts of the world, including most countries in the European Union (EU), Latin America, Israel and Russia, metamizole is the most popular non-opioid first-line analgesic and is sometimes even available over-the-counter. On the other hand, in some countries, e.g. the United States, the United Kingdom, Canada, Australia, Japan, Sweden, Denmark, Finland, and India it is not even on the (human) market. In 1977, the U.S. Food and drug administration (FDA) removed approval for human products and required that marketing of the drug for companion animals cease in 1995 to stop use in food animals. In 2019, the FDA has approved Zimeta^®^ (dipyrone injection) for the control of fever (pyrexia) in horses, but not for such intended for human consumption or food-producing animals, including lactating dairy animals, due to safety concerns for humans [7]. In Canada, metamizole is registered for use only on small animals and horses, whereas in the EU in contrast, metamizole preparations are approved for indications in food-producing animals (horses, cattle, swine) against disease states in which a positive influence by the analgesic, antipyretic, and spasmolytic effect of metamizole is to be expected. Due to Table 1 of the Commission Regulation (EU) No 37/2010 [8], the current Maximum Residual Limit (MRL) for metamizole in food producing cattle is 100 µg/kg each in fat, liver, muscle and kidneys, respectively, and 50 µg/kg in milk. The latency in cattle before slaughtering is 12 days for consumable tissue (IV) and 4 days for the dispensing of milk (IV), respectively. (…)

Additionally, within the discussion we adressed this aspect as follows:

(…) While metamizole is not uniformly available worldwide for food-producing animals due to the known human agranulocytosis risk, but a recent survey from Germany including neutropenia showed a rare risk rate of only 1:1602 [28]. It was suggested that the metamizole-induced agranulocytosis in humans was an immunologically mediated response and that stimulation of lymphocytes was a prerequisite . The Comittee for veterinary medical products concluded that the overall risk of agranulocytosis to humans from the ingestion of residues from treated animals was negligible [25]. Furthermore, data generated to support the approval of Zimeta^®^ by the U.S. FDA [7] did not indicate that agranulocytosis occurred in horses treated with the drug. Therefore, we considered metamizole as a very suitable add-on drug for our calves. (…)

Line 132-134. Was the surgery time 52 minutes in each animal? If not, some measure of variability in surgery time should be provided (± SD).

Yes, it was exactly 52 minutes in each animal.
Line 164-166. Mean is generally paired with std deviation. Median is generally paired with range?

Thank you very much, you are right, we corrected it into „Median“.

Line 136 – 153. Was there any interference between 4-MAA, 4-AA and meloxicam, xylazine, ketamine (chromatography, MS)? Why not use deuterated compound for IS?

The question is of course justified whether the other substances have an influence on the detection of 4-MAA/4-AA. Certainly, however, the spectra are so far apart from each other that clear measurements of 4-MAA/4-AA have taken place. Further studies would be required to assess that aspect in detail. 4-MAA, 4-AA analyses were performed in the Medical Laboratory Bremen (MVZ Medizinisches Labor Bremen GmbH, Bremen, Germany). Clonidine was used there as an internal standard.

Line 222-223 – AUC is correctly represented as ug*mL/hour?

Of course, you are right, the correct unit should be µg/mL*hour. We changed it in the manuscript.

I suggest that it would have been prudent to use half of the calves to receive metamizole and the other half do not get the same meloxicam.

The primary intention of our study was to improve the intraoperative analgesia for the calves by add-on metamizole to our standard anesthetic protocol in the Clinic for Ruminants of the Ludwig-Maximilians University of Munich (Germany). Therefore, we focussed only on a single dose of metamizole during surgery. Data from a saline control group without metamizole (but pure meloxicam) are shown within our first publication.

The non-steroidal anti-inflammatory drug (NSAID) meloxicam was primarily given to produce post-operative analgesia as post-operative pain results from prostaglandin-induced inflammatory processes in response to surgical trauma [54]. Although it was already applicated pre-emtively, a sufficient release of surgery-related inflammatory pain by NSAIDs is not clear [2].

Of course, we agree that a control group without pre-surgical meloxicam (only given before recovery) could have been advantageous due to assessment of pure metamizole effects. Unfortunatelly, we are not able to add it subsequently.

I would also suggest the authors attempt to shorten the discussion to make the paper clearer (emphasizing the important points?
For example:
Line 205-208 – How do the authors know the Cmax occurred at different time point in calves vs. dairy cows? Were the routes of administration the same? Was the dose of drug the same? It is clear that the drug used in each study was not the same, and interactions may occur?

The EMEA [25] cited data of a study in dairy cows, that got a single intravenous dose of a combination product containing butylscopolaminium bromide and metamizole at a dose rate corresponding to 22.6 mg metamizole per kg. Blood samples were taken from 0.25 to 72 hours after treatment. Maximum plasma concentration of 4-MAA were found in the samples taken 0.5 hours after treatment (mean 303 µg/mL).

Of course, you are right, there are obvious differences between our study and that study cited by EMEA: dairy cows vs. calves, and combination product containing 22.6 metamizole/kg bw vs. pure metamizole preparation dosed at 40 mg/kg. However, comparable commonalities between the studies are: both ruminants (cows/calves), single dose of metamizole given i.v., and blood samples taken from 0.25 to 72 hours after treatment. We definitely agree that pharmacokinetic interactions can occur in a combination product, as it was already reported by Aupanun et al. [19] and Giorgi et al. [21]. They observed higher 4-MAA plasma concentration using a pure metamizole preparation compared to a study using a combination product.

However, after we have revised the discussion completely, that part was almost removed:

(…) The recommended metamizole dose for cattle by the manufacturers is 20 - 40 mg/kg every 8 hours, applicated slowly IV. The elimination half-life of 4-MAA in anesthetized calves of 9.48 hours verified that supposed duration of analgesic action of at least 8 hours [13] after a single dose of metamizole. Compared with our anesthetized calves, the elimination half-life in humans (1.6–3.6 hours), but also in sheep (1.45-3 hours), goats (0.72 hours), horses (3.34 hours) or donkeys (1.81 hours) is much shorter [18,19,21,24]~~[1,14,15,17]~~. However, it is important to only compare study results using the same administration route due to a prolonged gradual drug release from the injection site to the vascular system after IM administration [18,20,21]. Additionally, it is important to only compare results based on pure metamizole formulations as combination products (e.g. Buscopan compositum^®^) can vary due to pharmacokinetic interactions between the two active compounds that can affect the metamizole metabolism or the 4-MAA kinetics [19,21]. (…)

Line 233-242. Please clarify: 303 ug/mL 4-MAA in calves vs. 101.62 ug/mL in adult cattle was a third (in calves) less than adult? Please clarify. If 4-MAA is only 1/3 of cows, then next sentence says 22.6 mg/kg vs. 40 mg/kg. Is this higher in calves and lower in cows or vice versa?

Thank you, but we have revised the discussion completely, that part was removed.

Line 239-241. What is the importance of higher drug level in calf than in adult cow? Neonates tend to be underdosed compared with adults. Depends upon species. Could also depend upon breed?

Thank you, but we have revised the discussion completely and removed this part.

There is much more information in the discussion that discredits this manuscript. Please shorten, provide concise information about calves. Spend small amount of time discussing importance of breed, age, sex etc. on PK of drug. The kinetics of drugs and their metabolites tells little about drug effect (without additional data). Consider this when describing the results etc.
I think one of the strengths of this paper is the clinical approach. However, judicious use of drugs is helpful. Further, having a control and test group would help?

Thank you very much for your assessment. We have thoroughly revised the manuscript again and have tried very hard to take up these suggestions.
However, we have not directly investigated the influence of breed, age and sex on PK of drugs. Our 4-MAA/4-AA data are preliminary in addition to clinical monitoring and plasma cortisol levels. In the discussion we included that aspect as follows:

(…) However, the relevance of differences in plasma peak concentration is limited without additional evidence of clinical efficacy because plasma concentrations need not automatically be proportional to it. Furthermore, C_max_ and T_max_ are not yet evidence of an inhibitory clinical effect above an analgesic threshold (minimum amount required to achieve pain relief) or therapeutic endpoint. Our data of T_max_ and elimination half-life seem to match with the clinical observations, as the behavior of 4-MAA fit best to a two-compartment model, which means that 4-MAA in calves is rapidly distributed into extravascular compartments, primarily highly perfused organs such as brain and liver. Fast 4-MAA distribution into the brain may account for a rapid onset of analgesia triggered by the release of ß-endorphin and synthesis of endocannabinoid [9]; distribution into the liver indicates metabolization by hepatic enzymes. Whereas 4-MAA accumulation in the brain is difficult to prove, increase of 4-AA plasma concentration indicates 4-MAA demethylation by enzymes of the hepatic cytochrome P450 system. Interestingly, generation of 4-AA occurred in five calves faster than in three other tested individuals suggesting individual differences in metabolic liver activity. Indeed, 4-MAA demethylases belong to the family of CYP2B and CYP3A enzymes, which are known for inter-individual variabilities in expression and activity in cattle [36,37]. Compared to adult cattle, however, 4-AA synthesis was overall slower in calves, which may be caused by immature activity of the two enzymes of the cytochrome P450 monooxygenase superfamily CYP2B and CYP3A [38]~~[31]~~, or substrate competition, as ketamine and xylazine are also CYP3A substrates [39]~~[32]~~. Human data show that meloxicam, or rather its 5-hydroxylation metabolite, is predominantly catalyzed by CYP2C9, with only minor contribution of CYP3A4 [40]~~[33]~~. Attributable activity and concentration differences in these biotransformation enzymes in the liver, specifically CYP2C9, have been identified in sheep, cattle, and goats [41]~~[34]~~. Generally, CYP enzymes are species-specific. In cattle, tissue-specific mRNA expression of different cytochrome P450 (CYP) isoforms could be proved. The absolute quantification of liver mRNAs showed that CYP3A38 was the most abundantly expressed CYP3A isoform in bovine liver, followed by CYP3A48. Conversely, CYP3A28 (corresponding to the abundant human CYP3A4) was expressed at levels <1% in different cattle breeds. Likewise to humans, physiological factors such as age, gender and breed have been shown to affect bCYP3A expression and/or activity [42]. Although we have not investigated the influence of age, gender and breed in our study, it is known from literature that breed causes more differences on drug metabolizing enzymes in calves than gender [43,44]. There was especially a faster elimination observed in pure milk than in pure meat production breeds influenced by muscle:body ratio. Our Simmental breed calves in comparison are a dual-purpose breed (well suited both for meat and milk production). (…)

Of course, we agree that a control group without pre-surgical meloxicam (only given before recovery) could have been advantageous due to assessment of pure metamizole effects. Unfortunatelly, we are not able to add it subsequently.

**Reviewer #2**: As there is not a lot of information in the literature on the pharmacokinetics of dipyrone/metamizole in cattle, this study has the potential to better inform clinical veterinary practice and NSAID decision-making. I have a few general and specific comments that may help improve the manuscript.
Thank you very much for your assessment. We have thoroughly revised the manuscript again and have tried very hard to improve it.

Title:
Since analgesia was not specifically evaluated in the study, it would be best to remove the word from the title. In the discussion, however, it would still be appropriate to speculate on the analgesic potential based on achievable plasma concentrations.
Thank you, we deleted it from the title, and revised the discussion section.

Abstract:
Line 42: It might be more appropriate to say that those metabolites were present, but there may be others that were not measured. The way the sentence currently reads suggests that those two metabolites are the only ones. Also, a better way to describe the model fit would be "observed data appeared to fit a two-compartment model best", rather than "corresponded to", since the method of analysis should be to attempt various models.
Thank you very much for your comment, we changed the sentence as follows:

The pharmacokinetic profile of 4-MAA appeared to fit a two-compartment model best. It was characterized by a fast distribution and slow elimination half-life (t_½alpha_ = 5.28 minutes, t_½beta_ = 9.48 hours).

Introduction:
It seems important to mention here and in the discussion section that this drug has been associated with agranulocytosis in humans and has been banned for use in food animals at various times in recent years in some countries. The food safety aspect of using it ought to be at least mentioned (e.g., is there an MRL/tolerance established, have withdrawal times been reported?). This is briefly mentioned in the introduction, but a bit more discussion might be appropriate, and naming the countries in which it is approved in food animals might be helpful to the reader. In addition, if approved in cattle, what indications were approved, and what dose, since this might help the reader put this study in context.
Since Reviewer 1 also wanted us to expand on this aspect, and also mention Zimeta^®^, which is approved for horses in the USA, we have expanded/changed this accordingly both in the introduction and in the discussion.

a) Introduction:

(…) In human medicine it was already launched in 1920 by the German Hoechst AG. But especially the rare risk of reversible but potentially fatal agranulocytosis led to the introduction of compulsory prescriptions for metamizole in Germany. Today, risk assessment and evaluation vary considerably from country to country. In many parts of the world, including most countries in the European Union (EU), Latin America, Israel and Russia, metamizole is the most popular non-opioid first-line analgesic and is sometimes even available over-the-counter. On the other hand, in some countries, e.g. the United States, the United Kingdom, Canada, Australia, Japan, Sweden, Denmark, Finland, and India it is not even on the (human) market. In 1977, the U.S. Food and drug administration (FDA) removed approval for human products and required that marketing of the drug for companion animals cease in 1995 to stop use in food animals. In 2019, the FDA has approved Zimeta^®^ (dipyrone injection) for the control of fever (pyrexia) in horses, but not for such intended for human consumption or food-producing animals, including lactating dairy animals, due to safety concerns for humans [7]. In Canada, metamizole is registered for use only on small animals and horses, whereas in the EU in contrast, metamizole preparations are approved for indications in food-producing animals (horses, cattle, swine) against disease states in which a positive influence by the analgesic, antipyretic, and spasmolytic effect of metamizole is to be expected. Due to Table 1 of the Commission Regulation (EU) No 37/2010 [8], the current Maximum Residual Limit (MRL) for metamizole in food producing cattle is 100 µg/kg each in fat, liver, muscle and kidneys, respectively, and 50 µg/kg in milk. The latency in cattle before slaughtering is 12 days for consumable tissue (IV) and 4 days for the dispensing of milk (IV), respectively. (…)

(…) The recommended metamizole dose for cattle by the manufacturers is 20 - 40 mg/kg every 8 hours, applicated slowly IV. (…)

b) Discussion:

(…) While metamizole is not uniformly available worldwide for food-producing animals due to the known human agranulocytosis risk, but a recent survey from Germany including neutropenia showed a rare risk rate of only 1:1602 [28]. It was suggested that the metamizole-induced agranulocytosis in humans was an immunologically mediated response and that stimulation of lymphocytes was a prerequisite . The Comittee for veterinary medical products concluded that the overall risk of agranulocytosis to humans from the ingestion of residues from treated animals was negligible [25]. Furthermore, data generated to support the approval of Zimeta^®^ by the U.S. FDA [7] did not indicate that agranulocytosis occurred in horses treated with the drug. Therefore, we considered metamizole as a very suitable add-on drug for our calves. (…)

Line 87: what dose was used in the previously published study?

In the previous study the same doses were used as in the current manuscript. That means 40 mg/kg of metamizole as well. We clarified it in the introduction:
The effects of metamizole on intraoperative nociception in the anesthetized calves are published elsewhere [26], which was investigated under the same conditions and using the same drug doses.

Methods:
Given the reports of agranulocytosis in people, it would be helpful if the investigators reported the reasons why CBCs were not performed at any time during the study, especially after drug administration.
Thank you for your comment. We included an explanation in the manuscript:

(…) CBCs without a differential were only examined when calves entered the clinic to ensure their suitability for the study. However, afterwards CBCs were not routinely performed if there was no concrete clinical suspicion. (…)

Even in human medicine, routine blood counts alone to monitor the leukocyte/granulocyte count during perioperative use of metamizole are not mandatory and should only be carried out in suspected cases with clinical symptoms like fever, exhaustion, sore throat and mucous membrane inflammation (such as stomatitis aphthosa, pharyngitis or tonsillitis, which ulcerate in the further course). It is known in humans that the risk increases with the duration of intake and decreases with distance from the last intake (after 10 days, no further increase in risk is to be expected). (Deutsches Ärzteblatt | Jg. 117 | Heft 4 | 24. Januar 2020). After only one single injection of metamizole and none known clear case in animals so far, no myelotoxic effect was expected in calves.

Line 147: Thank you for including LOD and LOQ. It would be helpful to note if the samples were all analyzed on the same day, and if not, was interday variability evaluated? This is not essential, but would be helpful for the reader.

Thank you very much for your comment. After blood sampling, serum was stored at -70 °C until further analysis. The samples were sent to the Medical Laboratory Bremen (MVZ Medizinisches Labor Bremen GmbH, Bremen, Germany) for analysis. The inter-assay variance is mapped by their running controls. These are typically in the following range:

 Interassay Variation in coefficient of variation in %

|  | Target low control µg/mL | Average found µg/mL  N= 16 | CV % | Target high control µg/mL | Average found µg/mL  N= | CV% |
| --- | --- | --- | --- | --- | --- | --- |
| 4-AA | 1 | 1,05 | 6,8 | 5 | 5,08 | 6,7 |
| 4-MAA | 1 | 1,02 | 7,1 | 5 | 4,8 | 7,4 |

Line 155: It would be appropriate for PK analysis to include attempts at fitting at least one- and two-compartment models as well as the method used to select which model. Please also clarify whether the parameters mentioned in the first sentence are from observed or modeled data. Were models attempted for the 4-AA data and they would not converge? It would be helpful to include that information, as an addition to explaining why only noncompartmental analysis was performed for this metabolite.
Thank you for your comment. These kinetic models for 4-MAA and 4-AA are described in the literature, e.g. Asmardi G, Jamali F. Pharmacokinetics of dipyrone in man; role of the administration route. Eur J Drug Metab Pharmacokinet. 1985;10: 121-125. doi: 10.1007/BF03189705. We included the explanation in the text as follows:

Based on that measured plasma concentrations of 4-MAA and 4-AA, the area under the curve (AUC), plasma peak concentration (C_max_) and time to reach C_max_ (T_max_) were calculated ~~determined~~ by using *PKSolver2.0* software, an add-in program for pharmacokinetic data analysis in Microsoft Excel [27]. Different models were attempted for the data but most of them did not converge. Only a two-compartment model adequately described the 4-MAA kinetics (R^2^ = 0.9975), or a noncompartmental analysis using the linear trapezoidal method correlated best with 4-AA kinetics, respectively.

Results:
The pharmacokinetic parameter data would be easier to review in tabular form. In addition, I am curious as to why the two-compartment model was described but the estimated parameters were not reported.
Of course, we could additionally give you the data in tabular form for the review. However, to our opinion, a figure shows it much clearer in the manuscript.

4-MAA (n=8):

4-AA (n=5, fast metabolizers):

4-AA (n=3, slow metabolizers):

Please excuse, if we could not fully comprehend your comment about the not reported estimated parameters of the two-compartment model as we have reported about t_1/2 alpha_ und t_1/2 beta_: (…) Subsequently, 4-MAA disappeared from the plasma in accordance with a two-compartment model, with a distribution half-life (t_½α_) of 5.28 minutes and an elimination half-life (t_½β_) of 9.48 hours. (…)

Additional consideration should be given as to the precision of the parameter estimates and significant figures. Reporting 100ths of hours, for example, is likely not meaningful and is an artifact of the modeling software and not the precision of the estimate.

Please excuse, if we could not fully comprehend your comment. Of course, we agree that the modelling software could give artifacts that could weaken the precision of the estimate. However, we only reproduced the data that we have. Of course, the parameters are all calculated and thus "estimated values". But the clinical plasma data served as their basis. We would be thankful, if you could please give us a more precise suggestion of your change request.

Discussion:
The discussion could do with a bit of reorganization to group like items closer together, for example, discussion of observed plasma concentrations and the observed PK parameters, followed by discussion of metabolites, and then the potential for drug interactions with the anesthetics. Also, I’m not sure comparing with non-ruminants is helpful if there are some data in ruminant species.
Thank you very much for your assessment. We have thoroughly revised the manuscript again and have tried very hard to take up these suggestions.

We agree, that it is much better to compare ruminants with ruminants. However, not every observation can be explained by findings in other ruminant studies as data situation is often very thin.

It is important to speculate on the effects on clearance and volume of distribution during anesthesia, particularly comparing with other drugs in cattle with and without the additional drugs.
Thank you, we included that aspect in the discusion as follows:

(…) Coetzee et al. [55] investigated the pharmacokinetics of intravenous and oral meloxicam in ruminant Holstein calves without additional anesthesia. After IV administration (0.5 mg/kg, the same dose as our calves were administered), meloxicam demonstrated a relatively small mean apparent volume of distribution at steady state (Vss) of 0.171 L/kg (0.15 - 0.19 L/kg) and a slow clearance (Cl) from the central compartment of 0.1 ml/min/kg (0.08 - 0.12 ml/kg/min). This resulted in a relatively long mean plasma terminal half-life of 20.35 hours (range, 17.84 to 22.76 hours). However, anesthesia and especially changes in cardiac output may affect not only the distribution of a drug but also its elimination clearance if it has a high hepatic extraction ratio. Changes in the pharmacokinetics of a drug resulting from changes in cardiac output may affect both early and steady-state arterial drug concentrations as well as its context-sensitive half-times [56]. For example, Waterman [5] could already prove in calves that after premedication with xylazine, whilst not affecting the half-lives significantly, reduced volumes of distribution and the clearance rate of ketamine and norketamine plasma concentrations by up to 50% (in females) compared to unpremedicated calves. As we have no control group of unanesthetized metamizole treated calves included in our study, our results are probaby not generally valid also for non-anesthetized animals. (…)

Line 206: The difference between 15 and 30 minutes is not likely to be meaningful, since samples were collected in either study only at those time points and not in between.
Thank you, we revised that part completely and we have deleted the probably somewhat irritating passage with the comparison between dairy cows and calves:

(…) In sheep, goats, horses and donkeys, the maximum 4-MAA concentration was rapidly detected a few minutes after IV metamizole application with a T_max_ of 0.08 hours [18,19,21,24]~~[1,14,15,17]~~. Assuming a similarly fast appearance of 4-MAA in calves, a greater plasma concentration and thus C_max_ at earlier time points are conceivable. As this scenario cannot be excluded, the C_max_ value determined in the present study after 15 minutes should be considered as a “first-point C_max_”. (…)

(…) However, the relevance of differences in plasma peak concentration is limited without additional evidence of clinical efficacy because plasma concentrations need not automatically be proportional to it. Furthermore, C_max_ and T_max_ are not yet evidence of an inhibitory clinical effect above an analgesic threshold (minimum amount required to achieve pain relief) or therapeutic endpoint. (…)

Line 212: It might be more appropriate to state that "it is assumed that the animals were not anesthetized and no other drugs were given at the same time".
Thank you, we revised that part completely and we have deleted the probably somewhat irritating passage with the comparison between dairy cows and calves.

Line 213: This sentence needs some clarification: are you suggesting there are drug-drug interactions? If so, perhaps be more explicit and provide more discussion than just the reference.
Thank you, we revised that part completely and we have deleted the probably somewhat irritating passage with the comparison between dairy cows and calves:

(…) Additionally, it is important to only compare results based on pure metamizole formulations as combination products (e.g. Buscopan compositum^®^) can vary due to pharmacokinetic interactions between the two active compounds that can affect the metamizole metabolism or the 4-MAA kinetics [19,21]. (…)

Line 223: Has the esterase been demonstrated to be the metabolic pathway in cattle? Perhaps this discussion should be more speculative given the lack of direct information, or additional evidence to support this based on the type of metabolite.
Thank you for your comment. You are absolutely right that the reference cited was in humans and not in cattle. The metabolic pathway in cattle is not explicitly demonstrated in literature. Therefore, we agree to discuss that point more speculatively:

(…) This is further supported by the hypothesis that the more acidotic the animal, the faster the esterase-dependent conversion could be, as in humans, the conversion of metamizole in 4-MAA depends on a non-enzymatic, pH-dependent esterase transformation [32]~~[22]~~. (…)

Line 310: As previously stated, care should be taken to make anything significant out of the differences in Cmax. In addition, the clinical relevance of any differences seems low, since the important parameter for analgesia is more likely to be time above an inhibitory concentration rather than a peak concentration.

Thank you very much for that comment, we changed the discussion as follows:

(…) In sheep, goats, horses and donkeys, the maximum 4-MAA concentration was rapidly detected a few minutes after IV metamizole application with a T_max_ of 0.08 hours [18,19,21,24]~~[1,14,15,17]~~. Assuming a similarly rapid onset of 4-MAA in calves, a higher plasma concentration and thus a Cmax at significantly earlier time points is conceivable. Since this scenario cannot be excluded, the Cmax value determined in the present study after 15 minutes should be considered as "first point Cmax". (…)

(…) However, the relevance of differences in plasma peak concentration is limited without additional evidence of clinical efficacy because plasma concentrations need not automatically be proportional to it. Furthermore, C_max_ and T_max_ are not yet evidence of an inhibitory clinical effect above an analgesic threshold (minimum amount required to achieve pain relief) or therapeutic endpoint. (…)

Line 318: CYP enzymes are species-specific, so caution should be taken to make that clear in this part of the discussion. What is known about the isoforms in cattle and what is speculation vs conclusive?
Thank you very much for this comment. We clarified that aspect in the manuscript as follows:

(…) Generally, CYP enzymes are species-specific. In cattle, tissue-specific mRNA expression of different cytochrome P450 (CYP) isoforms could be proved. The absolute quantification of liver mRNAs showed that CYP3A38 was the most abundantly expressed CYP3A isoform in bovine liver, followed by CYP3A48. Conversely, CYP3A28 (corresponding to the abundant human CYP3A4) was expressed at levels <1% in different cattle breeds. Likewise to humans, physiological factors such as age, gender and breed have been shown to affect bCYP3A expression and/or activity [42]. Although we have not investigated the influence of age, gender and breed in our study, it is known from literature that breed causes more differences on drug metabolizing enzymes in calves than gender [43,44]. There was especially a faster elimination observed in pure milk than in pure meat production breeds influenced by muscle:body ratio. Our Simmental breed calves in comparison are a dual-purpose breed (well suited both for meat and milk production). (…)

Line 337: Description of the methods of analgesia determination in the previous study, with perhaps more details about the conclusions, should be at least briefly included here. In addition, there are not sufficient data or analysis in this study to define a clear PK-PD relationship, so this should be quite speculative, and the impact of the other drugs used on analgesia has not been clearly delineated.
Thank you very much. We agree that this study is difficult to classify – it is no clear PK-PD study and no clear analgesia study on ist own, because it was an additional, preliminary data collection in few calves out of 26 animals.

To clarify that points, we completely revised the discussion:

(…) Those eight calves were part within a more comprehensive study design including 26 animals in total. Data of clinical monitoring and plasma cortisol levels from all the 26 animals, including saline control group without metamizole, are already published [26]. The additional investigation of 4-MAA and 4-AA should help to clarify whether these putative main analgesic metabolites of metamizole are formed at all in anesthetized calves because of not yet fully developed metabolic pathways and/or possible interactions with co-applied drugs. However, the merit of our study to perform the metabolic behavior of metamizole in a realistic clinical setting is at the same time a limitation of the study, because of missing control group without pre-surgically administered meloxicam. Therefore, our kinetic data in calves should be classified as preliminary without comparison, based on which further research could be planned. (…)

(…) In the overarching study design in which our calves were integrated, first the clinical monitoring parameters (especially heart rate and mean arterial blood pressure) were investigated intraoperatively in comparison with or without metamizole add-on. To better verify the unconscious nociceptive reaction to painful stimuli during surgery, plasma cortisol levels as an indicator of nociception were additionally measured - a common approach to assess pain/pain-related stress in bovine medicine [29]. Briefly summarized, the already published [26] plasma cortisol concentrations (PCC) showed no significant difference in baseline values before surgery (p = 0.84). During surgery a general cortisol release could be observed in all animals, but compared to saline controls (CG), the increase of PCC was delayed and consistently lower in metamizole-treated calves (MG). Afterwards, PCC remained elevated in the CG, whereas at 150 minutes after skin incision a significant decrease was observed for MG (MG: 11.6 (8.4 - 16.5) versus CG: 39.1 (27.3 - 81.4) nmol/L; p = 0.0026). Overall, the mean PCC in MG was 10.9 nmol/L lower than that in CG (p = 0.01). After 8.5 hours, cortisol levels equaled again in both groups (CG: 19.71 ± 16.51 nmol/L; MG: 15.98 ± 7.88 nmol/L). These results fit with the observed preliminary metabolic data (elimination half-life and C_max_ of 4-MAA and 4-AA) of metamizole. The mentioned general cortisol release probably had non-pain related reasons as isoflurane, xylazine, and also the placement of calves in dorsal recumbency may elevate plasma cortisol level [2,30,31]. (…)

(…) While an analgesic effect of 4-MAA by inhibition of prostaglandin synthesis is likely in calves, an interference with pre-applied COX inhibitor meloxicam is negligible as meloxicam inhibits COX activity by competing with the arachidonic acid binding side, whereas 4-MAA by sequestering COX stimulating radicals [53]. As expected, pre-applied meloxicam did not prevent 4-MAA synthesis in calves in principle as 4-MAA generation occured within 15 minutes after application. The non-steroidal anti-inflammatory drug (NSAID) meloxicam was primarily given to produce post-operative analgesia as post-operative pain results from prostaglandin-induced inflammatory processes in response to surgical trauma [54]. Although it was already applicated pre-emtively, a sufficient release of surgery-related inflammatory pain by NSAIDs is not clear [2]. (…)

(…) Our already published clinical monitoring data [26] showed that mean arterial pressure (MAP) significantly increased during anesthesia, independent of metamizole administration. This may have resulted from a waning in the hypotensive action of xylazine [58]. Heart rate (HR) initially decreased in almost parallel courses with and without metamizole until the beginning of the ‘suturing of the peritoneum and fascia’ period (29 minutes after skin incision), probably related to the depressive casrdiovascular effect of ketamine-xylazine, before it remained relatively constant until end of surgical intervention with metamizole add-on. The CG in contrast showed an increase afterwards.

Figures:
Drug concentration data and the visual assessment of linearity is more easily done when the x-axis is complete rather than broken. Consideration might also be given to a log10 scale for the y-axis, again for assessment of linearity.

Yes, we agree and thus now present our data with complete x axis.

As suggested, we used also log10 scale for our data and revealed (semi-)linearity for 4-MAA and 4-AA generation for fast metabolizers, but not slow metabolizers (see figures below).

 According to Ludden (1991), one of the more important sources of nonlinearity is the partial saturation of presystemic metabolism and has also been shown before for verapamil, propranolol and hydralazine. Whether this mechanisms also accounts for 4-AA in the slow metabolizing animal group remains to be determined. Although this would help to interpret our findings, it would be out of scope of the present study. Moreover, the calves are no longer available for testing potential differences in metabolism such as CYP enzyme activities, so that using data presentation with log10 scale would not bring further explanations than already being speculated and mentioned in the current manuscript. We would thus prefer our figures shown as they currently are.

Ludden, T. M. (1991). *Nonlinear Pharmacokinetics. Clinical Pharmacokinetics, 20(6), 429–446.* doi:10.2165/00003088-199120060-00001

x-axis complete	+ **y-axis in log**

 

6. PLOS authors have the option to publish the peer review history of their article ([what does this mean?](https://journals.plos.org/plosone/s/editorial-and-peer-review-process#loc-peer-review-history)). If published, this will include your full peer review and any attached files.


If you choose “no”, your identity will remain anonymous but your review may still be made public.


**Do you want your identity to be public for this peer review?** For information about this choice, including consent withdrawal, please see our [Privacy Policy](https://www.plos.org/privacy-policy).

Reviewer #1: No

Reviewer #2: No

[NOTE: If reviewer comments were submitted as an attachment file, they will be attached to this email and accessible via the submission site. Please log into your account, locate the manuscript record, and check for the action link "View Attachments". If this link does not appear, there are no attachment files.]

While revising your submission, please upload your figure files to the Preflight Analysis and Conversion Engine (PACE) digital diagnostic tool, <https://pacev2.apexcovantage.com/>. PACE helps ensure that figures meet PLOS requirements. To use PACE, you must first register as a user. Registration is free. Then, login and navigate to the UPLOAD tab, where you will find detailed instructions on how to use the tool. If you encounter any issues or have any questions when using PACE, please email PLOS at [figures@plos.org](mailto:figures@plos.org). Please note that Supporting Information files do not need this step.
