# Supplementary material for: Pharmacokinetics of metamizole (dipyrone) as an add-on in calves undergoing umbilical surgery
Source: PLoS One. 2022 Mar 15;17(3):e0265305. doi: 10.1371/journal.pone.0265305 (PMC8923478; doi:10.1371/journal.pone.0265305)
Supplement: Attachment — Submitted filename: Response to Reviewers.docx [file pone.0265305.s002.docx]

PONE-D-20-36269R1
Pharmacokinetics of metamizole (dipyrone) used as an add-on in calves undergoing umbilical surgery
PLOS ONE

Dear Dr. Baumgartner,

Thank you for submitting your manuscript to PLOS ONE. After careful consideration, we feel that it has merit but does not fully meet PLOS ONE’s publication criteria as it currently stands. Therefore, we invite you to submit a revised version of the manuscript that addresses the points raised during the review process.

Thank you for your efforts in revising the manuscript. Both reviewers and I acknowledge that the manuscript has been much improved. Nevertheless, there are still a few important points raised by reviewer #2 that would need to be addressed before a recommendation for acceptance can be made. Please, as suggested by the reviewer, also enlist the help of a colleague with experience in academic English language. While the grammar and syntax of the manuscript is overall correct, there are instances were language results awkward (probably as a result of direct translation from German to English). PLoS One does not provide language editing during typesetting and, therefore, I strongly suggest to have this taken care of now.

Please submit your revised manuscript by Sep 11 2021 11:59PM. If you will need more time than this to complete your revisions, please reply to this message or contact the journal office at [plosone@plos.org](mailto:plosone@plos.org). When you're ready to submit your revision, log on to <https://www.editorialmanager.com/pone/> and select the 'Submissions Needing Revision' folder to locate your manuscript file.

Please include the following items when submitting your revised manuscript:

- A rebuttal letter that responds to each point raised by the academic editor and reviewer(s). You should upload this letter as a separate file labeled 'Response to Reviewers'.
- A marked-up copy of your manuscript that highlights changes made to the original version. You should upload this as a separate file labeled 'Revised Manuscript with Track Changes'.
- An unmarked version of your revised paper without tracked changes. You should upload this as a separate file labeled 'Manuscript'.

If you would like to make changes to your financial disclosure, please include your updated statement in your cover letter. Guidelines for resubmitting your figure files are available below the reviewer comments at the end of this letter.

If applicable, we recommend that you deposit your laboratory protocols in protocols.io to enhance the reproducibility of your results. Protocols.io assigns your protocol its own identifier (DOI) so that it can be cited independently in the future. For instructions see: <http://journals.plos.org/plosone/s/submission-guidelines#loc-laboratory-protocols>. Additionally, PLOS ONE offers an option for publishing peer-reviewed Lab Protocol articles, which describe protocols hosted on protocols.io. Read more information on sharing protocols at <https://plos.org/protocols?utm_medium=editorial-email&utm_source=authorletters&utm_campaign=protocols>.

We look forward to receiving your revised manuscript.

Kind regards,

Angel Abuelo, DVM, MRes, MSc, PhD, DABVP (Dairy), DECBHM
Academic Editor
PLOS ONE


[Note: HTML markup is below. Please do not edit.]

Reviewers' comments:

Reviewer's Responses to Questions

**Comments to the Author**

1. If the authors have adequately addressed your comments raised in a previous round of review and you feel that this manuscript is now acceptable for publication, you may indicate that here to bypass the “Comments to the Author” section, enter your conflict of interest statement in the “Confidential to Editor” section, and submit your "Accept" recommendation.

Reviewer #1: All comments have been addressed

Reviewer #2: (No Response)

2. Is the manuscript technically sound, and do the data support the conclusions?

The manuscript must describe a technically sound piece of scientific research with data that supports the conclusions. Experiments must have been conducted rigorously, with appropriate controls, replication, and sample sizes. The conclusions must be drawn appropriately based on the data presented.

Reviewer #1: Yes

Reviewer #2: Partly

3. Has the statistical analysis been performed appropriately and rigorously?

Reviewer #1: Yes

Reviewer #2: Yes

4. Have the authors made all data underlying the findings in their manuscript fully available?

The [PLOS Data policy](http://www.plosone.org/static/policies.action#sharing) requires authors to make all data underlying the findings described in their manuscript fully available without restriction, with rare exception (please refer to the Data Availability Statement in the manuscript PDF file). The data should be provided as part of the manuscript or its supporting information, or deposited to a public repository. For example, in addition to summary statistics, the data points behind means, medians and variance measures should be available. If there are restrictions on publicly sharing data—e.g. participant privacy or use of data from a third party—those must be specified.

Reviewer #1: Yes

Reviewer #2: Yes

5. Is the manuscript presented in an intelligible fashion and written in standard English?

PLOS ONE does not copyedit accepted manuscripts, so the language in submitted articles must be clear, correct, and unambiguous. Any typographical or grammatical errors should be corrected at revision, so please note any specific errors here.

Reviewer #1: Yes

Reviewer #2: No

6. Review Comments to the Author

Please use the space provided to explain your answers to the questions above. You may also include additional comments for the author, including concerns about dual publication, research ethics, or publication ethics. (Please upload your review as an attachment if it exceeds 20,000 characters)

Reviewer #1: Thank you for addressing my concerns. A very exhaustive revision that should greatly improve the overall study.

Dear Jeffrey Lakritz,

thank you very much for your sympathetic assessment. We are very pleased to have met your expectations with our revision.

Reviewer #2: Because of the extensive revisions to the manuscript, it was difficult to evaluate only the revised portions, so I read it as if it were a new submission. It's possible, therefore, that there may be some repeated comments or suggestions. I make the following suggestions and comments to hopefully strengthen this submission.

Dear Reviewer #2,

thank you very much for your helpful comments. Hopefully, we are also able to meet your expectations after the current revision of the manuscript.

LANGUAGE: I recommend that the authors have the manuscript reviewed and edited by a native English speaker, as there are instances of awkward phrasing or inappropriate word use (for example, pain is not "released" by an analgesic, it is eliminated or reduced by one).

We are happy to comply with your request and have had the manuscript professionally edited by the American Journal Experts (AJE) (see certificate attached). We hope that the linguistic improvement now meets your expectations.

GENERAL COMMENTS: As previously recommended by a reviewer, this manuscript should tighten up the focus to a straight forward description of the pharmacokinetics of two of the known metabolites of metamizole. It should also reduce the amount of speculation in the discussion about analgesia, pharmacodynamics, and metabolic differences among populations of cattle, given that this investigation did not evaluate any of those. A small amount of speculation is certainly warranted, e.g., plasma concentrations reported in other species to be analgesic, or CYP450 isoforms associated with differences in metabolism, but they should be short and succinct. In addition, the impact of the sampling times on the parameter estimates is not discussed at all, and there is a possibility that they might have impacted the results and their interpretation.

We tried to shorten the discussion, especially the named parts. We ask for your understanding that we have done this moderately without any major loss of content in order to be able to continue to maintain the wishes of Reviewer #1.

We included the impact of the sampling times on the parameter estimates.

OTHER SPECIFIC SUGGESTIONS:
ABSTRACT: One cannot describe two Cmax's - there is only one "highest" which is what Cmax represent. In addition, the time of Cmax is properly called Tmax, rather than conflating the two parameters into a single report with time and concentration. Describing two peaks is reasonable, and speculating about the reason is as well, but they are not both Cmax.

Thank you very much for that valuable input, we corrected it in the text.

Line 91 and others: Please use the phrase withdrawal time, as it is understood among cattle health professionals what that means. (Avoid "latency" or "waiting period".)
Thank you very much for that note, we changed it in the manuscript.

Line 195: One cannot collect blood with EDTA and extract serum - please clarify whether plasma or serum was used to analyze drug concentrations.
Thank you for your comment and please excuse our carelessness. It was indeed studied plasma. We corrected it in the text.

Line 212/PK analysis: More details about the two-compartment model selected are needed (even if it's just to describe the model number within the software), as well as why it was selected - it's hard to believe one-compartment didn't converge at all, but perhaps the AIC and visual examination of the residuals suggested two-compartment was a better fit? In addition, noncompartmental analysis only describes what's there, so there is no correlation needed to determine if it's appropriate.

Pharmacokinetic parameters were deduced from plasma concentration-time data using *PKSolver2.0* software, which allows compartmental and non-compartmental analyses of the experimental data. Visual inspection of the curve, residual analysis and Akaike's information criterion estimation (for 4-MAA 41.99) were used to choose the model best fitting the data. All data points were weighted by the inverse square of the fitted value.

Different models were attempted for the data and a two-compartment model (CA, IV bolus, 2 compartment) best described the 4-MAA kinetics (R2 = 0.9975). A non-compartmental analysis (IV bolus) using the linear trapezoidal method was used for calculating 4-AA kinetics parameter. We concluded the two-compartment situation for following reasons:

1. Observed 4-MAA plasma concentration data indicate a biexponential decline, which let us suggest a kinetic described by a two-compartment model.

2. Calculation of kinetic parameter by using PK Solver 2.0 showed that observed plasma data corresponds well to predicted data when using the model “CA, IV bolus, 2 compartment” (Zhang et al., 2010). See figure below.

3. PK Solver 2.0 further assessed data by examination of semilogarithmic plots of the serum concentration-time curves, which again indicated that a two-compartment model would be adequate. See figure below.

4. 4-MAA-Kinetik follows a two-compartment model after IV bolus application also in other species, e.g., humans (Asmardi G, Jamali F. Pharmacokinetics of dipyrone in man; role of the administration route. Eur J Drug Metab Pharmacokinet. 1985;10: 121-125. doi: 10.1007/BF03189705.) and horses (Klaus AM, Schlingloff Y, Kleinitz U, Böttcher M, Hapke HJ. Pharmacokinetic study of dipyrone metabolite 4-MAA in the horse and possible implications for doping control. J Vet Pharmacol Ther. 1997;20: 204-208. doi: 10.1111/j.1365-2885.1997.tb00096.x). Additionally, it was used in a study in calves for intravenous meloxicam PK, for example (Cagnardi P, Guccione J, Villa R, D'Andrea L, Di Loria A, Ferrante MC, et al. Clinical efficacy and pharmacokinetics of meloxicam in Mediterranean buffalo calves (*Bubalus bubalis*). PLoS ONE 2017;12: e0187252. doi: 10.1371/journal.pone.0187252).

Line 223/Results - PK parameters are best provided in a table, and there are some parameters missing from the results, such as the micro-constants. One cannot evaluate PK just from figures representing serum concentrations, so tabular data is expected. In addition, given the length of the estimated half-lives, and that the duration of sample collection was only 24 hours, the authors will want to speculate in the discussion about whether samples were collected for long enough to actually effectively estimate half-life and other parameters (was extrapolation to infinity possible for example).

As requested, we provide our data in a table (including AUC extrapolated to infinity):

**Table 1. Main pharmacokinetic parameters of 4-methylaminoantipyrine (4-MAA) and 4-aminoantipyrine (4-AA) following single intravenous administration of metamizole (40 mg/kg) in calves.** Values are means.

| **Parameter** | **4-MAA**  **(n=8)** | **4-AA**  **(fast metabolizers, n=5)** | **4-AA**  **(slow metabolizers, n=3)** |
| --- | --- | --- | --- |
| R² | 1.00 |  |  |
| Lambda z (1/hour) |  | 0.08 | 0.11 |
| t_1/2alpha_ (hours) | 0.088 (5.29 minutes) |  |  |
| t_1/2beta_ (hours) | 9.49 | 8.87 | 6.23 |
| T_max_ (hours) | 0.25 | 0.25 | 6.50 |
| C_max_ (µg/mL) | 101.63 | 2.66 | 1.66 |
| K10 (1/hour) | 0.88 |  |  |
| K12 (1/hour) | 6.40 |  |  |
| K21 (1/hour) | 0.65 |  |  |
| C0 (µg/mL) | 500.64 | 2.79 | 0.10 |
| Vss (mg/(µg/mL)) | 17.76 | 249.19 | 283.36 |
| V1 (mg/(µg/mL)) | 1.65 |  |  |
| CL1 ((mg)/(μg/mL)/hour) | 1.45 | 19.40 | 28.18 |
| V2 (mg/(µg/mL)) | 16.11 |  |  |
| CL2 ((mg)/(μg/mL)/hour) | 10.55 |  |  |
| Vz ((mg)/(μg/mL)) |  | 248.30 | 253.45 |
| AUC_0–t_ (µg/mL*hour) | 482.06 | 35.87 | 26.97 |
| AUC_0–∞_ (µg/mL*hour) | 570.64 | 42.53 | 29.28 |
| AUMC_0–∞_ (µg/mL*hour²) | 7010.28 | 546.25 | 294.38 |
| MRT (hours) | 12.28 | 12.84 | 10.06 |

R², correlation coefficient; Lambda z, terminal phase rate constant; t_1/2alpha_, distribution half-life; t_1/2beta_, terminal elimination half-life; T_max_, time of peak; C_max_, peak plasma concentration; K10, rate at which the drug leaves the system from the central compartment (elimination rate); K12, rate at which the drug passes from central to peripheral compartment; K21, rate at which the drug passes from peripheral to central compartment; C0, serum concentration at time 0; Vss, volume of distribution at steady-state; V1, volume of distribution in the central compartment; CL1, clearance of the central compartment; V2, volume of distribution in the peripheral compartment; CL2, clearance of the peripheral compartment; Vz, volume of distribution based on the terminal phase; AUC_0–t_, area under the plasma concentration–time curve; AUC_0–∞_, area under the plasma concentration–time curve extrapolated to infinity; AUMC_0–∞_, area under the first moment curve from zero to infinity; MRT, mean residence time.

Of course, sampling time points may have an influence on the results and their interpretation. In our study, samples were taken immediately before, 15 and 30 minutes after metamizole administration and then every half an hour for 24 hours. In contrast, in other studies e.g., samples were taken every 15 minutes within the first hour, but only every 2 hours within the first day afterward [18-21]. In sheep, another sample was added after 36 hours [18], and in pigs after 48 and 72 hours [20]. However, in both studies metamizole was also (or only) applied IM, which was expected a longer metabolization than IV. Concerning the length of 4-MAA and 4-AA half-lives in calves, sampling time could have been longer in our study, but extrapolation to infinity was possible.

7. PLOS authors have the option to publish the peer review history of their article ([what does this mean?](https://journals.plos.org/plosone/s/editorial-and-peer-review-process#loc-peer-review-history)). If published, this will include your full peer review and any attached files.


If you choose “no”, your identity will remain anonymous but your review may still be made public.


**Do you want your identity to be public for this peer review?** For information about this choice, including consent withdrawal, please see our [Privacy Policy](https://www.plos.org/privacy-policy).

Reviewer #1: **Yes:** Jeffrey Lakritz

Reviewer #2: No

[NOTE: If reviewer comments were submitted as an attachment file, they will be attached to this email and accessible via the submission site. Please log into your account, locate the manuscript record, and check for the action link "View Attachments". If this link does not appear, there are no attachment files.]

While revising your submission, please upload your figure files to the Preflight Analysis and Conversion Engine (PACE) digital diagnostic tool, <https://pacev2.apexcovantage.com/>. PACE helps ensure that figures meet PLOS requirements. To use PACE, you must first register as a user. Registration is free. Then, login and navigate to the UPLOAD tab, where you will find detailed instructions on how to use the tool. If you encounter any issues or have any questions when using PACE, please email PLOS at [figures@plos.org](mailto:figures@plos.org). Please note that Supporting Information files do not need this step.
