# Supplementary material for: Pharmacokinetics of metamizole (dipyrone) as an add-on in calves undergoing umbilical surgery
Source: PLoS One. 2022 Mar 15;17(3):e0265305. doi: 10.1371/journal.pone.0265305 (PMC8923478; doi:10.1371/journal.pone.0265305)
Supplement: Attachment — Submitted filename: Response to Reviewers.docx [file pone.0265305.s003.docx]

PONE-D-20-36269R2

Pharmacokinetics of metamizole (dipyrone) as an add-on in calves undergoing umbilical surgery

PLOS ONE

Dear Dr. Baumgartner,

Thank you for submitting your manuscript to PLOS ONE. After careful consideration, we feel that it has merit but does not fully meet PLOS ONE’s publication criteria as it currently stands. Therefore, we invite you to submit a revised version of the manuscript that addresses the points raised during the review process.

Please address the comments of reviewer #1 through another round of revisions.

Please submit your revised manuscript by Dec 12 2021 11:59PM. If you will need more time than this to complete your revisions, please reply to this message or contact the journal office at [plosone@plos.org](mailto:plosone@plos.org). When you're ready to submit your revision, log on to <https://www.editorialmanager.com/pone/> and select the 'Submissions Needing Revision' folder to locate your manuscript file.

Please include the following items when submitting your revised manuscript:

- A rebuttal letter that responds to each point raised by the academic editor and reviewer(s). You should upload this letter as a separate file labeled 'Response to Reviewers'.
- A marked-up copy of your manuscript that highlights changes made to the original version. You should upload this as a separate file labeled 'Revised Manuscript with Track Changes'.
- An unmarked version of your revised paper without tracked changes. You should upload this as a separate file labeled 'Manuscript'.

If you would like to make changes to your financial disclosure, please include your updated statement in your cover letter. Guidelines for resubmitting your figure files are available below the reviewer comments at the end of this letter.

If applicable, we recommend that you deposit your laboratory protocols in protocols.io to enhance the reproducibility of your results. Protocols.io assigns your protocol its own identifier (DOI) so that it can be cited independently in the future. For instructions see: <https://journals.plos.org/plosone/s/submission-guidelines#loc-laboratory-protocols>. Additionally, PLOS ONE offers an option for publishing peer-reviewed Lab Protocol articles, which describe protocols hosted on protocols.io. Read more information on sharing protocols at <https://plos.org/protocols?utm_medium=editorial-email&utm_source=authorletters&utm_campaign=protocols>.

We look forward to receiving your revised manuscript.

Kind regards,

Angel Abuelo, DVM, MRes, MSc, PhD, DABVP (Dairy), DECBHM
Academic Editor
PLOS ONE

Journal Requirements:

Please review your reference list to ensure that it is complete and correct. If you have cited papers that have been retracted, please include the rationale for doing so in the manuscript text, or remove these references and replace them with relevant current references. Any changes to the reference list should be mentioned in the rebuttal letter that accompanies your revised manuscript. If you need to cite a retracted article, indicate the article’s retracted status in the References list and also include a citation and full reference for the retraction notice.


[Note: HTML markup is below. Please do not edit.]

Reviewers' comments:

Reviewer's Responses to Questions

**Comments to the Author**

1. If the authors have adequately addressed your comments raised in a previous round of review and you feel that this manuscript is now acceptable for publication, you may indicate that here to bypass the “Comments to the Author” section, enter your conflict of interest statement in the “Confidential to Editor” section, and submit your "Accept" recommendation.

Reviewer #1: (No Response)

Reviewer #2: All comments have been addressed

2. Is the manuscript technically sound, and do the data support the conclusions?

The manuscript must describe a technically sound piece of scientific research with data that supports the conclusions. Experiments must have been conducted rigorously, with appropriate controls, replication, and sample sizes. The conclusions must be drawn appropriately based on the data presented.

Reviewer #1: Partly

Reviewer #2: Yes

3. Has the statistical analysis been performed appropriately and rigorously?

Reviewer #1: No

Reviewer #2: Yes

4. Have the authors made all data underlying the findings in their manuscript fully available?

The [PLOS Data policy](http://www.plosone.org/static/policies.action#sharing) requires authors to make all data underlying the findings described in their manuscript fully available without restriction, with rare exception (please refer to the Data Availability Statement in the manuscript PDF file). The data should be provided as part of the manuscript or its supporting information, or deposited to a public repository. For example, in addition to summary statistics, the data points behind means, medians and variance measures should be available. If there are restrictions on publicly sharing data—e.g. participant privacy or use of data from a third party—those must be specified.

Reviewer #1: Yes

Reviewer #2: Yes

5. Is the manuscript presented in an intelligible fashion and written in standard English?

PLOS ONE does not copyedit accepted manuscripts, so the language in submitted articles must be clear, correct, and unambiguous. Any typographical or grammatical errors should be corrected at revision, so please note any specific errors here.

Reviewer #1: No

Reviewer #2: Yes

6. Review Comments to the Author

Please use the space provided to explain your answers to the questions above. You may also include additional comments for the author, including concerns about dual publication, research ethics, or publication ethics. (Please upload your review as an attachment if it exceeds 20,000 characters)

Reviewer #1: PONE-D-20-36269R2 - Pharmacokinetics of metamizole (dipyrone) as an add-on in calves undergoing umbilical surgery

Line 70-72. Please cite references for this comment:
“However, the rare risk of reversible but potentially fatal 71 agranulocytosis led to the introduction of compulsory prescriptions for metamizole in Germany.”
I would want to know if residues in animals may be harmful to consumers

Thank you for your comment. We included a reference here (Line 72) and also a sentence in this paragraph (Line 91-94) concerning your point of interest.

We already had explained this point in more detail in the discussion to show why we chose metamizole as an add-on:

See Line 311-320: While metamizole is not uniformly available worldwide for food-producing animals due to the known human agranulocytosis risk, a recent survey from Germany including neutropenia showed a rare risk rate of only 1:1602 [29]. It was suggested that metamizole-induced agranulocytosis in humans is an immunologically mediated response and that stimulation of lymphocytes is a prerequisite. The Committee for Veterinary Medical Products concluded that the overall risk of agranulocytosis to humans from the ingestion of residues from treated animals was negligible [10]. Furthermore, data generated to support the approval of Zimeta^®^ by the U.S. FDA [8] did not indicate that agranulocytosis occurred in horses treated with the drug. Therefore, we considered metamizole as a very suitable add-on drug for our calves.

Additionally, for your interest, please see the following detailed explanation of The European Agency for the Evaluation of Medicinal Products (EMEA). Committee for Veterinary Medicinal Products. Metamizole. Summary Report (2). EMEA/MRL/878/03_FINAL. **2003**. Available from: <https://www.ema.europa.eu/en/documents/mrl-report/metamizole-summary-report-2-committee-veterinary-medicinal-products_en.pdf>:

Line 132-133. Please revise this sentence. As written, the meaning is nonsensical

“The clinical, veterinary research study was conducted between August 2013 and July 2014, except for wintertime, during which the temperature was too low.”
Indicate which months were excluded and also please define why temperature is too low (cold enough to result in damage to the active ingredient?)

Perhaps the sentence failed our meaning. When the outdoor temperature dropped below freezing (late October 2013 to mid-March 2014), the execution of the experiments was interrupted. The main reason for this was the husbandry of calves in igloos after surgery/anesthesia. The primary concern here was not the measurement of metabolites, but rather perioperative management and body temperature regulation (hypothermia).

We revised the sentence as follows (Line 137-141):

The clinical, veterinary research study was conducted between August 2013 and July 2014. When the outside temperature dropped below freezing (end of October 2013 to mid-March 2014), the performance of the experiments was interrupted because of the increased risk of perioperative adverse events (mainly hypothermia).

Line 28-29. Please explain why you could not evaluate drug in young animals that are anesthetized. Since these are milk fed animals with hay, TMR, with mineral lick availability (TMR does not include minerals?). Pre-ruminants may have markedly different disposition of this drug when compared to ruminants? Including a statement regarding quantity of hay/TMR consumed daily may help.

Please excuse us if our answer does not quite meet your intentions. There must have been a mistake in the line numbers and we are not quite sure which line you are referring to.

Of course, we were able to investigate metamizole in the young anesthetized animals. However, in the introduction we speculated about the comparability of the results, that it might well make a pharmacokinetic difference when immature calves were also given other substances in parallel compared to literature data from lactating, adult, awake dairy cows. We did not refer to the influence of the feed, but to the possibly still immature metabolism and a possible interaction with co-applied substances.

For your interest: The animals received fresh whole milk heated to 43 °C three times a day (7:00 am, 12:00 noon, 6:30 pm). The daily watering ration of the animals was continuously increased to 15% of the body mass until the start of the experiment, but it amounted to a maximum of 3.5 liters of whole milk per milk meal. All animals were provided **ad libitum** with a total mixed ration consisting of 50% corn silage, 25% calf grain and 25% calf muesli, as well as a mineral lick and hay. Straw bedding and water were renewed twice daily. Assessment and documentation of feeder intake was performed by the animal caretakers according to clinic internal guidlines.

We had already described this in the text as follows (see Line 152-154): „The calves were fed whole milk three times a day and had **free access** to water, hay, a total mixed ration (50% corn silage, 25% calf grain and 25% calf muesli), and mineral licks.” Accordingly, we would like to leave the sentence as it is and have only added the composition of TMR in brackets for explanatory purposes.

Line 146-150. I think I would indicate all calves were purchased to repair hernia? Then state all were healthy during the study?

We did not claim that the animals were completely healthy, but:

„On the day of purchase, a short clinical examination by a veterinarian showed no health disorders apart from uncomplicated umbilical hernia.” (see Line 144-146). (…) “The inclusion criteria were based on clinically unremarkable findings by a veterinarian and an undisturbed general condition of the animal, including feed intake and blood analysis (complete blood count (CBC) without a differential) one day before surgery. The exclusion criteria were any signs of disease other than an uncomplicated hernia.” (see Line 154-158).

We feel that this statement is true and, accordingly, we would like to leave it as is.

Line 162-166. It would be prudent to indicate whether drug was administered by the same catheter as blood sampling for analysis of metamizole metabolites. If 2 catheters placed, then indicate so in methods section.
In fact only one catheter was used for blood sampling and drug injection. Before and after each blood sampling or drug administration through the jugular venous catheter, it was flushed again with isotonic saline, its correct position was checked, and the neck region was checked for non-irritation. We have included that in the text accordingly (see Line 175-178).

For a blood sample collection, the first 2 ml of blood was discarded after flushing bevor the samples were drawn (as already described in Line 204-206).

Line 190-210. I think you should consider providing accuracy, precision for both individual runs as well as between day runs. Linearity is another consideration.

Samples were analyzed by the Medical Laboratory Bremen (MVZ Medizinisches Labor Bremen GmbH, Bremen, Germany). This laboratory has accreditation by DIN EN ISO 15189, which includes the evaluation of assay precision.

While we are on subject of quantification, how does one determine T1/2 elimination, clearance, Vd etc of metabolite(s) after injection of the parent drug?

As mentioned in M+M, blood samples were analyzed for the pharmacological active metabolites by HPCL/LC-MS. Based on plasma concentration, pharmacokinetic parameters were determined by using *PKSolver2.0* software. This fact is now included into the M+M section.

How does one get Vss of 17.76 from the 5 rapid and 3 slow metabolizers (249.19 + 283.36/2)?

We do not show that rapid metabolizers have a Vss of 17.76, and slow metabolizers 249.19 + 283.36/2 as you mentioned here. We show in Tabl.1 that 4-MAA has a Vss of 17.76, and that 4-AA has a Vss of 249.19 in rapid, and 283.36 in slow metabolizers. Thus, these different Vss values apply for different metabolites, not for fast/slow metabolizers.

If the Volumes for metabolites is so large, why does clearance increase for slow metabolizers. Clearance and volume of distribution are two independent parameters and are subject to different inter-individual parameters. A correlation between volume of distribution and clearance is thus not possible.

Why is Vss units of mg/(ug/mL)

Vss is the apparent volume of distribution at steady state. It can be calculated as follows:

**Vss = A(t) (mg) / C(t) (mg/L)**

A(t) represents the amount of drug in the body at time = t

C(t) represents plasma concentration of the drug at time = t

Consequently, Vss unit is mg/(mg/L) corresponding mg/(µg/mL)

Line 285-289. I am confused about the stated goals of this study. Apparently you have data on plasma cortisol concentrations after metamizole and surgery/anesthesia. I do not see how you can comment on this without having controls. It is very confusing you did not specifically address the changes in PCC with metamizole in calve undergoing anesthesia and surgery. Without your stated controls, this study seems woefully incomplete.

We are sorry that apparently, despite our efforts to be as transparent as possible, the connection between our two studies was not entirely clear. We would like to try to explain the aim of our study to you again and how it was conducted:

The original aim was a study with calves in the Clinic for Ruminants of the LMU Munich, in which the routine anesthesia protocol valid until then was to be improved with regard to analgesia by metamizole add-on. This was evaluated in the context of a doctoral thesis (Magdalena Behrendt-Wippermann "Significance of the administration of metamizole in the preemptive pain management of surgical procedures on the navel of calves"). **A total of 26 calves were included in the original study. The study was conducted double-blinded.**

A trial lasted for a total of 9 days: intensive preoperative, intraoperative, and postoperative monitoring of the animals took place one day before surgery (D-1), on the day of surgery (D 0), and one day after surgery (D+1). On D-1 through the seventh day (D+7) post-surgery, daily body mass and feeder intakes were documented.

On D-1 and D 0, as well as on D+1, the doctoral student assessed the calves' pain perception using a pain questionnaire. First, an assessment of the animal's general pain was made using a visual analog scale (VAS). Next, both the animal's response to the presence of the doctoral student and palpation of the umbilical region and the swelling and redness of the surgical area were assessed using a numeric pain scale. Simultaneously, the rectal temperature (RT) of the experimental animal and the external temperature were recorded.

Video recordings were made at D-1 and D 0, as well as at D+1 for video-assisted behavioral observation of the animals without additional measurement equipment. In addition, the animals wore a chest strap for simultaneous measurement of heart rate (HR) and heart rate variance (HRV).

On D-1, the animal was also weighed and venous access was established under xylazine sedation. At D 0, baseline blood levels were drawn after meloxicam administration (plasma cortisol concentration), or again after skin incision at 5, 30, 60, 150, and 510 minutes, and at 2.5 and 8.5 hours. One hour before skin incision, **metamizole administration or (blinded) the same amount of saline was given to a control group**. The following parameters were documented close-meshed during anesthesia: Respiratory rate (RR), reflexes, bulb position, end-tidal CO2 concentration (etC02), and isoflurane concentrations, body temperature, HR, pulse, peripheral oxygen saturation, and arterial blood pressure. Arterial blood gases were also monitored. **The experimental procedure was strictly standardized**.

**Data on clinical monitoring and plasma cortisol concentration (PCCs) from all 26 animals, including the saline control group without metamizole, have already been published [27].**

**Plasma from 8 animals from these 26 was additionally sent to the laboratory in Bremen for determination of the major metabolites of metamizole, 4-MAA and 4-AA. These are now the subject of the current manuscript,** as unfortunately it was not possible to include these data into our first publication. Therefore, they appear here as a separate manuscript probably "incomplete". **Therefore we have always referred to the already published data**, because they have been generated under the same conditions in the same animals.

Reviewer #2: (No Response)

7. PLOS authors have the option to publish the peer review history of their article ([what does this mean?](https://journals.plos.org/plosone/s/editorial-and-peer-review-process#loc-peer-review-history)). If published, this will include your full peer review and any attached files.


If you choose “no”, your identity will remain anonymous but your review may still be made public.


**Do you want your identity to be public for this peer review?** For information about this choice, including consent withdrawal, please see our [Privacy Policy](https://www.plos.org/privacy-policy).

Reviewer #1: No

Reviewer #2: No

[NOTE: If reviewer comments were submitted as an attachment file, they will be attached to this email and accessible via the submission site. Please log into your account, locate the manuscript record, and check for the action link "View Attachments". If this link does not appear, there are no attachment files.]

While revising your submission, please upload your figure files to the Preflight Analysis and Conversion Engine (PACE) digital diagnostic tool, <https://pacev2.apexcovantage.com/>. PACE helps ensure that figures meet PLOS requirements. To use PACE, you must first register as a user. Registration is free. Then, login and navigate to the UPLOAD tab, where you will find detailed instructions on how to use the tool. If you encounter any issues or have any questions when using PACE, please email PLOS at [figures@plos.org](mailto:figures@plos.org). Please note that Supporting Information files do not need this step.
