# Supplementary material for: Pharmacokinetics of metamizole (dipyrone) as an add-on in calves undergoing umbilical surgery
Source: PLoS One. 2022 Mar 15;17(3):e0265305. doi: 10.1371/journal.pone.0265305 (PMC8923478; doi:10.1371/journal.pone.0265305)
Supplement: Attachment — Submitted filename: Response to Reviewers.docx [file pone.0265305.s004.docx]

PONE-D-20-36269R3

Pharmacokinetics of metamizole (dipyrone) as an add-on in calves undergoing umbilical surgery

PLOS ONE

Dear Dr. Baumgartner,

Thank you for submitting your manuscript to PLOS ONE. After careful consideration, we feel that it has merit but does not fully meet PLOS ONE’s publication criteria as it currently stands. Therefore, we invite you to submit a revised version of the manuscript that addresses the points raised during the review process.

In line with the reviewer's comment, you must provide the details/results of the method validation in the manuscript, even if the analyses were conducted at an external laboratory.

Please submit your revised manuscript by Feb 04 2022 11:59PM. If you will need more time than this to complete your revisions, please reply to this message or contact the journal office at [plosone@plos.org](mailto:plosone@plos.org). When you're ready to submit your revision, log on to <https://www.editorialmanager.com/pone/> and select the 'Submissions Needing Revision' folder to locate your manuscript file.

Please include the following items when submitting your revised manuscript:

- A rebuttal letter that responds to each point raised by the academic editor and reviewer(s). You should upload this letter as a separate file labeled 'Response to Reviewers'.
- A marked-up copy of your manuscript that highlights changes made to the original version. You should upload this as a separate file labeled 'Revised Manuscript with Track Changes'.
- An unmarked version of your revised paper without tracked changes. You should upload this as a separate file labeled 'Manuscript'.

If you would like to make changes to your financial disclosure, please include your updated statement in your cover letter. Guidelines for resubmitting your figure files are available below the reviewer comments at the end of this letter.

If applicable, we recommend that you deposit your laboratory protocols in protocols.io to enhance the reproducibility of your results. Protocols.io assigns your protocol its own identifier (DOI) so that it can be cited independently in the future. For instructions see: <https://journals.plos.org/plosone/s/submission-guidelines#loc-laboratory-protocols>. Additionally, PLOS ONE offers an option for publishing peer-reviewed Lab Protocol articles, which describe protocols hosted on protocols.io. Read more information on sharing protocols at <https://plos.org/protocols?utm_medium=editorial-email&utm_source=authorletters&utm_campaign=protocols>.

We look forward to receiving your revised manuscript.

Kind regards,

Angel Abuelo, DVM, MRes, MSc, PhD, DABVP (Dairy), DECBHM
Academic Editor
PLOS ONE

Journal Requirements:

Please review your reference list to ensure that it is complete and correct. If you have cited papers that have been retracted, please include the rationale for doing so in the manuscript text, or remove these references and replace them with relevant current references. Any changes to the reference list should be mentioned in the rebuttal letter that accompanies your revised manuscript. If you need to cite a retracted article, indicate the article’s retracted status in the References list and also include a citation and full reference for the retraction notice.

[Note: HTML markup is below. Please do not edit.]

Reviewers' comments:

Reviewer's Responses to Questions

**Comments to the Author**

1. If the authors have adequately addressed your comments raised in a previous round of review and you feel that this manuscript is now acceptable for publication, you may indicate that here to bypass the “Comments to the Author” section, enter your conflict of interest statement in the “Confidential to Editor” section, and submit your "Accept" recommendation.

Reviewer #1: All comments have been addressed

2. Is the manuscript technically sound, and do the data support the conclusions?

The manuscript must describe a technically sound piece of scientific research with data that supports the conclusions. Experiments must have been conducted rigorously, with appropriate controls, replication, and sample sizes. The conclusions must be drawn appropriately based on the data presented.

Reviewer #1: Partly

3. Has the statistical analysis been performed appropriately and rigorously?

Reviewer #1: I Don't Know

4. Have the authors made all data underlying the findings in their manuscript fully available?

The [PLOS Data policy](http://www.plosone.org/static/policies.action#sharing) requires authors to make all data underlying the findings described in their manuscript fully available without restriction, with rare exception (please refer to the Data Availability Statement in the manuscript PDF file). The data should be provided as part of the manuscript or its supporting information, or deposited to a public repository. For example, in addition to summary statistics, the data points behind means, medians and variance measures should be available. If there are restrictions on publicly sharing data—e.g. participant privacy or use of data from a third party—those must be specified.

Reviewer #1: Yes

5. Is the manuscript presented in an intelligible fashion and written in standard English?

PLOS ONE does not copyedit accepted manuscripts, so the language in submitted articles must be clear, correct, and unambiguous. Any typographical or grammatical errors should be corrected at revision, so please note any specific errors here.

Reviewer #1: Yes

6. Review Comments to the Author

Please use the space provided to explain your answers to the questions above. You may also include additional comments for the author, including concerns about dual publication, research ethics, or publication ethics. (Please upload your review as an attachment if it exceeds 20,000 characters)

Reviewer #1: It would be appropriate to provide method validation details for this type of study (linearity, within and between day variability, stability, etc.)

Even though we thought we had already presented the required method validation details, we would like to present them to you again in a clear tabular form and have also included the following tabular overview in the manuscript:

| Internal standard | Clonidin |
| --- | --- |
| intra-assay coefficient of variation, CV % (N=9) | 4-AA: 2.5 %  4-MAA: 2.0 % |
| LOQ (limit of quantification; S/N=10) | 4-AA: 0.1 µg/mL  4-MAA: 0.1 µg/mL |
| LOD (limit of detection; S/N=3) | 4-AA: 0.02 µg/mL  4-MAA: 0.04 µg/mL |
| Linear ranges | 4-AA: 0.1 - 20 µg/mL  4-MAA: 0.1 - 50 µg/mL |
| Method | LC-MS/MS |
| Material | Serum or plasma |
| Stability | 3 weeks at 2 - 8 °C |

The inter-assay variance is mapped by the running controls of the Medical Laboratory Bremen (MLHB). These are typically in the following range:

Interassay Variation in coefficient of variation in %:

|  | Target low control µg/mL | Average found µg/mL  N= 16 | CV % | Target high control µg/mL | Average found µg/mL  N= 16 | CV % |
| --- | --- | --- | --- | --- | --- | --- |
| 4-AA | 1 | 1,05 | 6,8 | 5 | 5,08 | 6,7 |
| 4-MAA | 1 | 1,02 | 7,1 | 5 | 4,8 | 7,4 |

**Please note:**

The suitability of LC-MS/MS methods is well approved for PK studies due to both its high selectivity and sensitivity as well as its quantitation capabilities. Although no in depth-validation on calf plasma has been performed and matrix effects cannot be fully excluded, it is very well acceptable to apply the human plasma-based method in a preliminary study. We are fully aware that a more detailed PK study requires a quantitation method dedicated to calf plasma as specimen. In the present work, this methodology was however not available and its development beyond the scope of the study.

**General information about the quality management of the** **Medical Laboratory Bremen (MLHB):**

The MLHB is a testing laboratory accredited by the German Accreditation Body for Chemistry (DACH) since August 15, 2001 according to DIN EN ISO/IEC 17025. Within the scope of the 2004 assessment, the accreditation was extended to include the requirements of DIN EN ISO 15189 Medical Laboratories - Requirements for Quality and Competence. On 28.01.2021, the MLHB was issued the current accreditation certificates according to DIN EN ISO/IEC 17025:2018 and DIN EN ISO/IEC 15189:2014 by the German Accreditation Body (DAkkS) after successful reaccreditation.

For more than 15 years, the MLHB has thus maintained a quality management system that complies with the requirements of the guidelines of the German Medical Association and supplements them in many points. Furthermore, the quality and competence of the MLHB are checked in regular expert assessments by the DAkkS and confirmed with the issuance of the accreditation certificate.

The MLHB has flexible accreditation in all areas of investigation, which allows procedures to be modified and new procedures to be established within the scope of accreditation, without prior assessment by the DAkkS.

In addition to accreditation according to DIN EN ISO 17025 and 15189, further quality certificates exist, for example for the field of "Endocrinology, Diabetes and Metabolism" by the UEMS.

7. PLOS authors have the option to publish the peer review history of their article ([what does this mean?](https://journals.plos.org/plosone/s/editorial-and-peer-review-process#loc-peer-review-history)). If published, this will include your full peer review and any attached files.


If you choose “no”, your identity will remain anonymous but your review may still be made public.


**Do you want your identity to be public for this peer review?** For information about this choice, including consent withdrawal, please see our [Privacy Policy](https://www.plos.org/privacy-policy).

Reviewer #1: No

[NOTE: If reviewer comments were submitted as an attachment file, they will be attached to this email and accessible via the submission site. Please log into your account, locate the manuscript record, and check for the action link "View Attachments". If this link does not appear, there are no attachment files.]

While revising your submission, please upload your figure files to the Preflight Analysis and Conversion Engine (PACE) digital diagnostic tool, <https://pacev2.apexcovantage.com/>. PACE helps ensure that figures meet PLOS requirements. To use PACE, you must first register as a user. Registration is free. Then, login and navigate to the UPLOAD tab, where you will find detailed instructions on how to use the tool. If you encounter any issues or have any questions when using PACE, please email PLOS at [figures@plos.org](mailto:figures@plos.org). Please note that Supporting Information files do not need this step.
